# A Novel Technology to Boost Natural Production of Hyaluronic Acid in the Skin Tissue: Human Histology Study

**DOI:** 10.1111/jocd.70159

**Published:** 2025-04-17

**Authors:** David J. Goldberg

**Affiliations:** ^1^ Skin Laser and Surgery Specialists Hackensack New Jersey USA; ^2^ Schweiger Dermatology Group New York City New York USA; ^3^ Icahn School of Medicine at Mt. Sinai New York City New York USA

**Keywords:** facial rejuvenation, histology, hyaluronic acid, monopolar radiofrequency, targeted ultrasound

## Abstract

**Introduction:**

Combining monopolar radiofrequency (RF) with targeted ultrasound (TUS), this study investigated whether these modalities promote facial rejuvenation through the production of hyaluronic acid (HA) in human skin.

**Methods:**

Seven subjects (51–64 years, BMI 21.1–29.8 kg/m^2^) were enrolled and divided into three treatment groups in this single‐center study; Group A (*n* = 3, simultaneous RF + TUS), Group B (*n* = 3, stand‐alone RF), and control (*n* = 1, no treatment). Both treated groups underwent four (4) 60‐min treatments on the face delivered 7–14 days apart. Punch biopsies (3 mm in diameter) were collected from the infra‐auricular area at baseline and both follow‐up visits and stained for HA by using hyaluronic acid binding protein. Digital photographs were taken to document changes in visual appearance. Finally, the subjects' comfort and satisfaction were assessed.

**Results:**

There was a statistically significant (*p* < 0.05) average increase at 1 month in the HA‐stained area of +112 358.7 μm^2^ in group A (RF + TUS) representing an increase of 48.65%. The treatment effect peaked at 3 months with an increase of +156 345.2 μm^2^, corresponding to a 67.69% increase in the HA‐stained area. In Group B, there was no significant difference in the average increase of the HA‐stained area between 1 month (+14 830 μm^2^) and 3 months (+20 995 μm^2^) corresponding to a 6.76% and 9.56% increase, respectively. The control samples did not indicate any changes throughout the study. Digital photographs of the RF + TUS group showed both a decrease in rhytids and tighter skin. Therapies were comfortable with no adverse events.

**Conclusion:**

Overall, this study has shown that the combination treatment of RF + TUS has a more pronounced and sustained effect on facial rejuvenation compared to RF alone. The measurable increase in the production of HA with simultaneous use of RF + TUS peaked at a 3‐month follow‐up, suggesting the gradual advancement of the treatment effect and overall improvement in facial appearance.

**Trial Registration:**

ClinicalTrials.gov identifier: NCT05987917

## 1 Introduction

Hyaluronic acid (HA) is a naturally occurring polysaccharide (glycosaminoglycan) that is abundantly present in human tissues, particularly the skin [1]. It has garnered significant attention as an ideal tool for facial rejuvenation due to its unique properties and versatility [1, 2]. Hyaluronic acid plays a crucial role in tissue hydration, lubrication, and cellular function [3]. It possesses exceptional water‐binding capabilities, allowing it to retain moisture and provide optimal hydration to the skin [3, 4]. This property makes it a key contributor to skin elasticity, texture, and overall skin quality.

**FIGURE 1:**
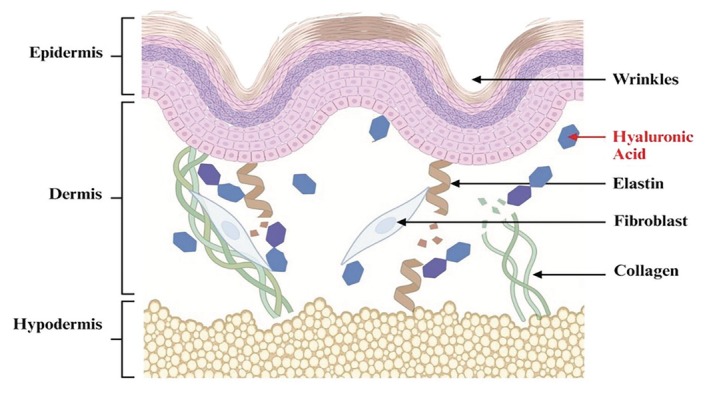
Diagram showing the structure of aging skin with significantly broken and reduced levels of HA (created with Biorender.com).

However, the natural aging process, in combination with environmental factors, can lead to a decline in HA levels. As HA levels decrease, skin may lose its plumpness, resulting in diminished facial volume and contour. This HA deficiency can lead to a range of skin‐related consequences, such as the appearance of wrinkles, fine lines, and volume loss (see Figure 1) on the skin [5]. Facial rejuvenation, therefore, has become increasingly popular in recent years, as individuals strive to achieve a more youthful and vibrant appearance [6].

Hyaluronic acid is commonly associated with dermal fillers or injection treatments that are now a common choice for addressing age‐related changes in areas such as the cheeks, nasolabial folds, and lips [7]. For example, HA fillers introduce synthetic HA into the skin, which binds to water and blends with the naturally present HA to enhance facial volume. These fillers use the ability of HA to hold volume and serve as facial volume implants which are usually delivered below the skin, sometimes even below muscles. However, such application is not focused on restoring and rejuvenating the depleted skin structure itself [8, 9]. Therefore, there is a compelling need for comprehensive and noninvasive technological solutions capable of actively promoting intrinsic alterations in the skin to address age‐related facial changes.

One such approach is a novel technology (EXION, BTL Industries Inc., Boston, MA) that simultaneously emits monopolar radiofrequency (RF) and targeted ultrasound (TUS) to enhance the production of HA. In prior veterinary studies, it was suggested that the concurrent application of monopolar RF in conjunction with TUS resulted in a notable increase in endogenous HA production. Furthermore, these two studies conducted a comparison of this technology with standalone RF and RF combined with non‐TUS [10]. Both of these alternative approaches did not appear to produce a significant impact on the synthesis of natural HA. It is hypothesized that this novel technology uses its ultrasound component to alter the impedance of the skin tissue, increase cell permeability, and, most importantly, deliver mechanical stimuli to the fibroblasts located in the reticular dermis [11, 12, 13]. The RF field creates an oscillating electrical current that induces collisions between charged ions and molecules in the skin, which in turn generates heat [14, 15]. The rise in temperature in the dermis initiates neocollagenesis and elastogenesis [16]. Additionally, the combination of RF and TUS results in greater HA production within the dermis, leading to significant improvements in facial rejuvenation.

Consequently, evaluating the histological changes associated with enhanced HA production is crucial for optimizing facial rejuvenation procedures [17]. By analyzing the dermal architecture and HA synthesis, researchers can objectively elucidate the mechanisms underlying the effectiveness of any facial therapy [18]. It can also allow for the identification of any potential structural alterations caused by any therapeutic intervention [19]. This study conducted a histological examination of the facial skin tissue before the treatment and posttreatment to assess the changes in HA levels resulting from simultaneous monopolar RF and TUS application. These results were then compared with facial skin tissue samples obtained from patients treated solely with RF and with those from one control subject.

This study aims to evaluate the clinical efficacy and safety of the therapy for facial rejuvenation through documentation of the changes in the skin tissue related to the levels of HA and to investigate whether the observed effects in previous veterinary studies can be replicated in human skin.

## 2 Methods

### 2.1 Study Population

This study was a single‐center, three‐arm, open‐label design. It was approved by the Advarra Institutional Review Board (IRB ID: Pro00049067) and followed the 1975 Declaration of Helsinki guidelines (ClinicalTrials.gov Identifier: NCT05987917).

Seven healthy subjects (51–64 years, BMI 21.1–29.8 kg/m^2^) were recruited and participated in this study. Written informed consent was obtained from all participating subjects based on inclusion (above 21 years old, BMI ≤ 35 kg/m^2^ and presence of clearly visible wrinkles in the treated area) and exclusion criteria (bacterial or viral infection, acute inflammations, impaired immune system, skin related autoimmune diseases, poor healing, or unhealed wounds in the treatment area).

The patients were randomly divided into three study groups—Group A, Group B, and Control, using a computer‐generated list and respecting the site of enrollment.

- Group A: *n* = 3, treated with a device simultaneously emitting RF and TUS, mean age 56.7 years, BMI 25.9 kg/m^2^, skin type II–IV.
- Group B: *n* = 3 treated with a device utilizing only RF energy, mean age 57 years, BMI 23.6 kg/m^2^, skin type II–III.
- Control: *n* = 1 did not receive any treatment, 58 years, BMI 25.3 kg/m^2^, skin type III.

### 2.2 Treatment Protocol

Group A was treated using the face applicator delivering the RF and TUS energies simultaneously (Group A). Group B was treated by standalone monopolar RF.

The subjects of study Groups A and B received four treatments of the entire face area, including the forehead, upper and lower cheeks (comprising infra‐auricular area as well), and the area around the eyes. Individual treatments were spaced by 7–14 days. The entire area was assessed for wrinkles and skin laxity reduction.

No anesthetic agents were used prior to or during treatment. A conductive gel was applied to the treatment area to ensure good energy flow. In accordance with the protocol and clinical guidelines, the RF intensity was adjusted based on patient tolerability and was set to 70% for each treatment.

Before treatment, all jewelry was removed and the skin was cleared of any cosmetic products. Subjects were provided with a headband to pull hair off their face and a black neck drape to conceal their clothing. Each treatment session lasted up to 60 min. The control subject did not receive any treatment.

### 2.3 Punch Biopsy

Punch biopsies of the skin tissue from the infra‐auricular area were performed at baseline, followed by 1‐ and 3‐month follow‐up visits. Subjects were put in a supine position, and a 1% lidocaine anesthesia was applied to the biopsied area prior to the sampling to minimize pain and discomfort.

Full‐thickness biopsies, measuring 3 mm in diameter, were taken from the subjects with a depth of 1.5–2.0 mm. Following the procedure, the biopsy wounds were carefully closed, disinfected, and closely monitored throughout the study for the healing process. The subjects were provided with comprehensive instructions for home wound care to ensure adequate and optimal healing.

The control samples were acquired from the face of the control subject at the corresponding time points (baseline—at least 1 week before the treatments, 1 and 3 months) in order to mirror the sampling regimen in the treated groups. Each participant provided three biopsy samples of facial skin tissue for the evaluation.

### 2.4 Histological Examination

The taken samples were processed for histological examination. The samples were fixed, embedded, cut, and stained by conventional methods using ethanol‐formalin‐glacial acetic acid (ethyl alcohol 70%, formalin 10% of 37% solution, water 15%, glacial acetic acid 5%).

Each biopsy sample was sectioned for staining and subsequent analysis employing routine procedures. Samples were specifically embedded in paraffin wax, sliced into 5‐μm‐thick slices, and stained for the visualization of HA and surrounding tissue structures under a microscope. All samples were labeled using the subject ID and the description of the corresponding visit to prevent any mix‐ups.

A clinical histologist analyzed the stained samples to determine the levels of HA, including any pathological changes, in order to rule out any acute or lasting skin damage resulting from the study treatment.

### 2.5 HA Staining and Imaging

**FIGURE 2:**
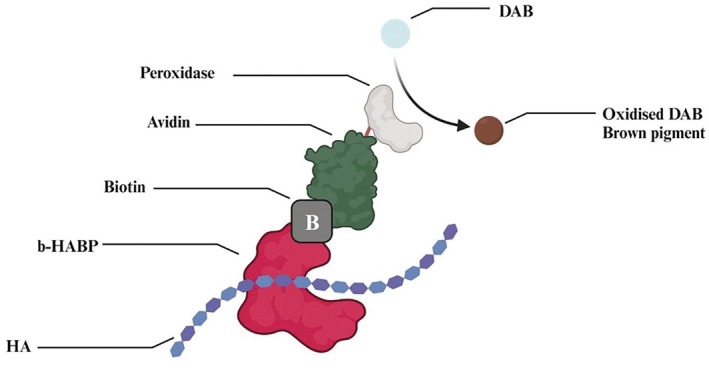
Diagram illustrating the histochemical approach of staining tissues and cells with biotinylated hyaluronic acid binding protein (b‐HABP) and diaminobenzidine (DAB). Peroxidase oxidizes DAB into a brown pigment that is easily visualized under microscopy (created with Biorender.com).

The staining mechanism (see Figure 2) employed conventional HA‐specific HABP and biotinylated‐HABP staining for the visualization of HA under light and confocal microscopy, which enabled a detailed visual assessment of HA and the general tissue structure. The HA in the samples was visualized by increased intensity of brown color.

For HA staining, a biotinylated HA‐binding protein (b‐HABP; Seikagaku Corp., Tokyo, Japan) at 5 μg/mL was added and incubated at 4°C overnight. The slides were washed with peripheral blood smear (PBS) before incubation with fluorescence avidin‐D (20 μg/mL; Vector Laboratories, Burlingame, CA) at room temperature for 1 h. After a final washing step, the stained samples were mounted with Cytoseal XYL Thermo Scientific microscopy slides. The slides were observed, analyzed, and photographed by confocal laser scanning microscope (TCS‐40; Leica Microsystems, Cambridge, UK).

Hyaluronic acid quantification involved using ImageJ software and applying semiautomatic segmentation in the hue–saturation–brightness (HSB) color system. The amount of HA was finally expressed in terms of the occupied area (square micrometers) in analyzed images.

### 2.6 Photography and Questionnaires

At the baseline visit, after the last (4th) treatment, at the 1‐ and 3‐month follow‐up visits, 2D and 3D digital photographs of the treated areas were taken from subjects in all groups.

A photographic imaging system LifeViz Mini (QuantifiCare S.A. France) was used to take 3D photographs from the left, right, and front views of the face at multiple angles. These images were used for therapy effectiveness assessment by three independent evaluators using the Global Aesthetic Improvement Scale (GAIS) and Fitzpatrick Wrinkle and Elastosis Scale (FWES). Before and after each visit, the examination for possible adverse effects was performed in all subjects.

The 5‐point Likert scale Subject Satisfaction Questionnaire (SSQ) was administered to the subjects from Groups A and B after the final treatment and at all follow‐up visits to assess their satisfaction with the therapy results according to this criteria: (5) *Strongly agree*, (4) *Agree*, (3) *Neither agree nor disagree*, (2) *Disagree*, and (1) *Strongly disagree*. The SSQ consisted of the following questions: *Q1: Wrinkles in the treatment area have been reduced after treatments; Q2: Skin laxity in the treated area has improved; Q3: Overall appearance of the skin has been improved after treatments; Q4: I am satisfied with the treatment results*.

To further document patient comfort during treatments, the Therapy Comfort Questionnaire (TCQ) was administered after the final treatment session in Groups A and B with a 5‐point Likert scale question. Subjects responded to the question; *Q1: I found the treatment comfortable* according to the same criteria as SSQ. The TCQ also included a Numerical Analog Scale (NAS), where pain scores ranged from 0—no pain, to 10—worst possible pain, experienced during the treatment.

### 2.7 Statistical Analysis

The descriptive statistic was calculated (mean and standard deviation). Student's *t*‐test and Friedman test were performed with a significance level set at α = 0.05 to show statistically significant differences between the groups.

## 3 Results

In total, seven subjects were enrolled in the study. Both treated groups (A and B) completed four treatments and two follow‐up visits. There was no withdrawal from the study.

Collected punch biopsies healed properly with no additional medical care needed. The histological examination showed no pathological abnormalities in the skin tissue. Digital photographs were taken to document changes in visual appearance while the subject's comfort and satisfaction were assessed.

There were no adverse events reported throughout the study, and no treatment‐related downtime was experienced by any subject.

### 3.1 Histology Examination

**FIGURE 3:**
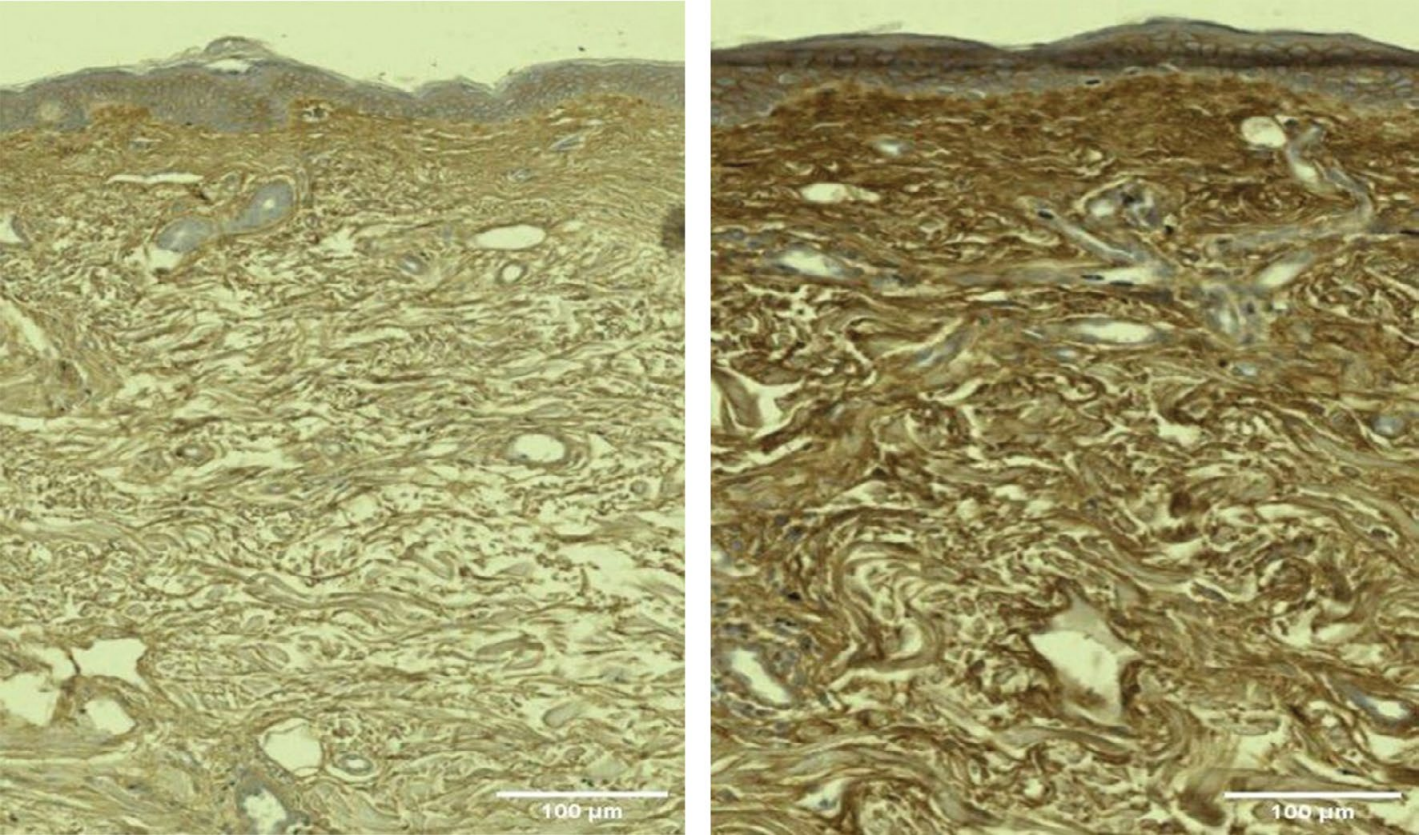
Histology examination visualized increased amount (darker brown color) of hyaluronic acid in a Group A (RF + TUS) subject: baseline (left) and 3‐month follow‐up (right). The connective tissue also became denser and better organized.

**FIGURE 4:**
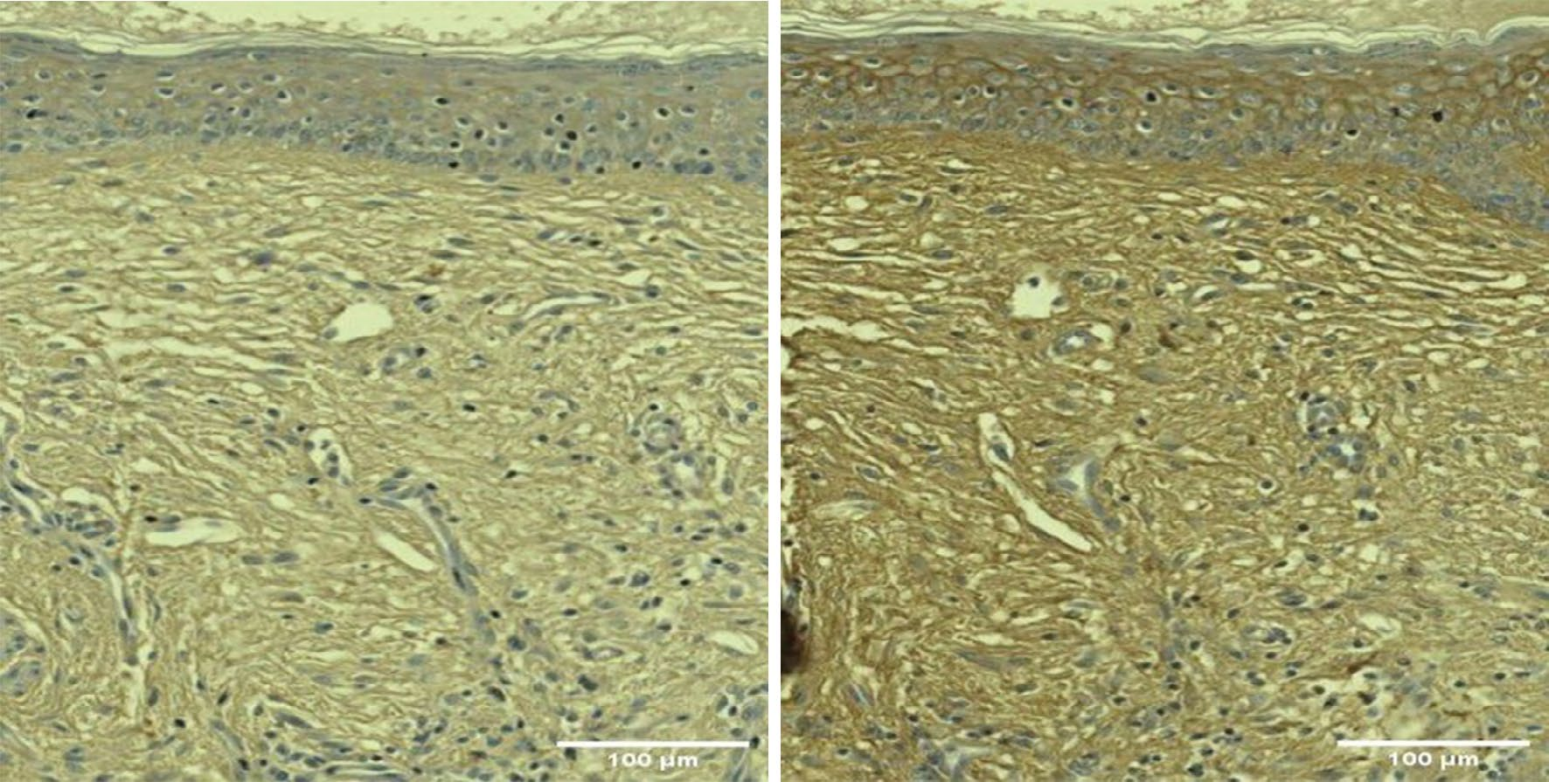
Histology examination visualized little amount (less brown color) of hyaluronic acid in a Group B (RF only) subject: baseline (left) and 3‐month follow‐up (right). The connective tissue was also less organized.

Histological examination revealed an increased amount of HA in posttreatment samples, with increased intensity and extent of HABP staining shown as the visualization of the brown color under microscopy (Figures 3 and 4).

**FIGURE 5:**
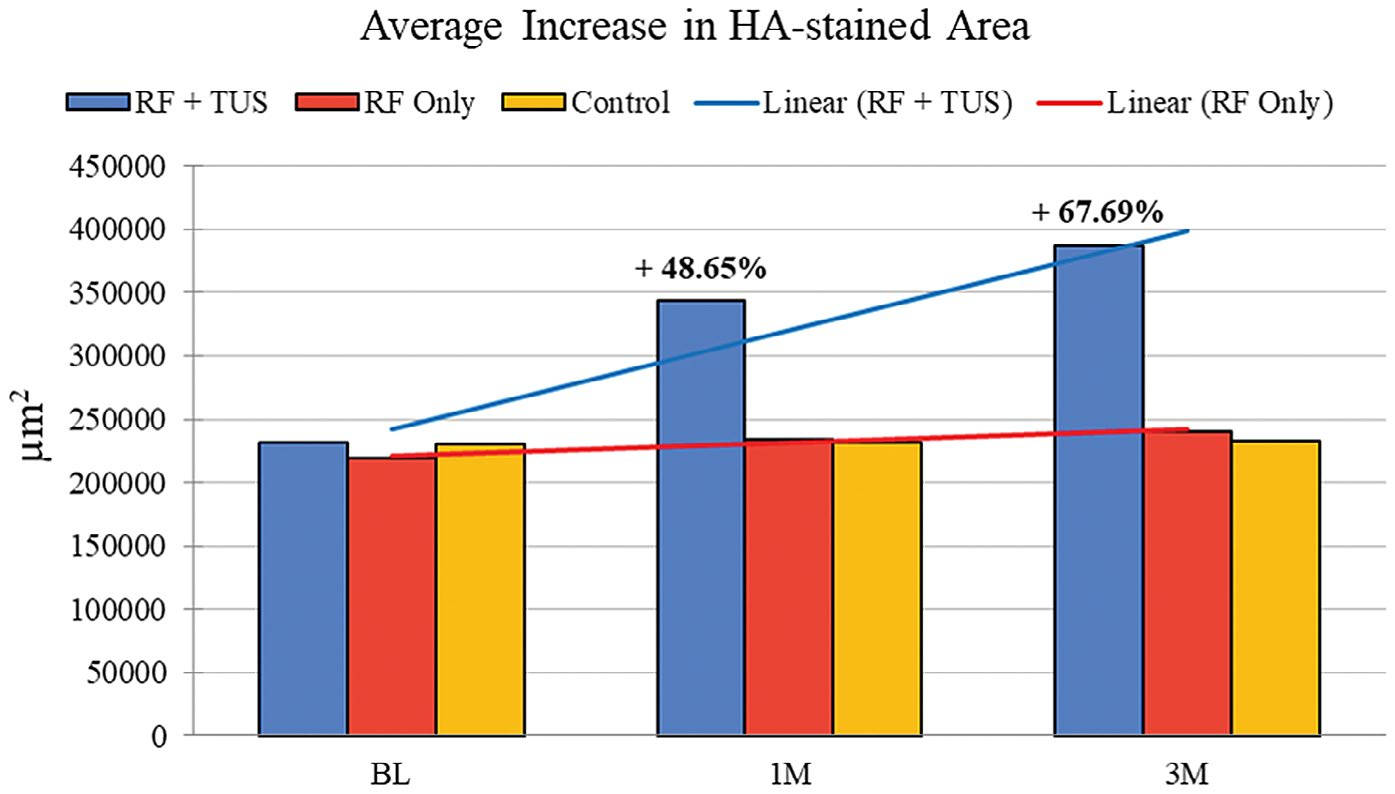
Average HA concentrations from subjects are shown, and statistical tests were performed for 1 and 3 months using the baseline for comparison. The statistically significant (*p* < 0.05) increase in HA‐stained area in Group A (blue) was from the combined effects of RF + TUS.

There was a statistically significant (*p* < 0.05) average increase at 1 month in the HA‐stained area of +112 358.7 μm^2^ in group A (RF + TUS) in Figure 5, representing an increase of 48.65%. The treatment effect peaked at 3 months with an increase of +156 345.2 μm^2^, corresponding to a 67.69% increase in the HA‐stained area (see Table 1).

**TABLE 1:** Histology examination for Group A (RF + TUS).

Parameters	BL	1 M	3 M
HA μm^2^	230 965.5	343 324.2	387 310.7
Difference μm^2^	N/A	112 358.7	156 345.2
Difference (%)	N/A	48.65%	67.69%

Abbreviations: 1 M, 1‐month follow‐up; 3 M, 3‐month follow‐up; BL, baseline; N/A, not available.

In Group B, there was an insignificant (*p*‐value = 0.07) difference in the average increase of HA‐stained area between 1 month (+14 830 μm^2^) and 3 months (+20 995 μm^2^) corresponding to a 6.76% and 9.56% increase, respectively (see Table 2).

**TABLE 2:** Histology examination for Group B (RF only).

Parameters	BL	1 M	3 M
HA μm^2^	219 233.7	234 054.0	240 219.4
Difference μm^2^	N/A	14 830	20 995.8
Difference (%)	N/A	6.76%	9.58%

Abbreviations: 1 M, 1‐month follow‐up; 3 M, 3‐month follow‐up; BL, baseline; N/A, not available.

There were no observable changes or increases in HA‐stained area indicated in the control samples at 1 and 3 months, as shown in Figure 5.

### 3.2 Photography Evaluation

**FIGURE 6:**
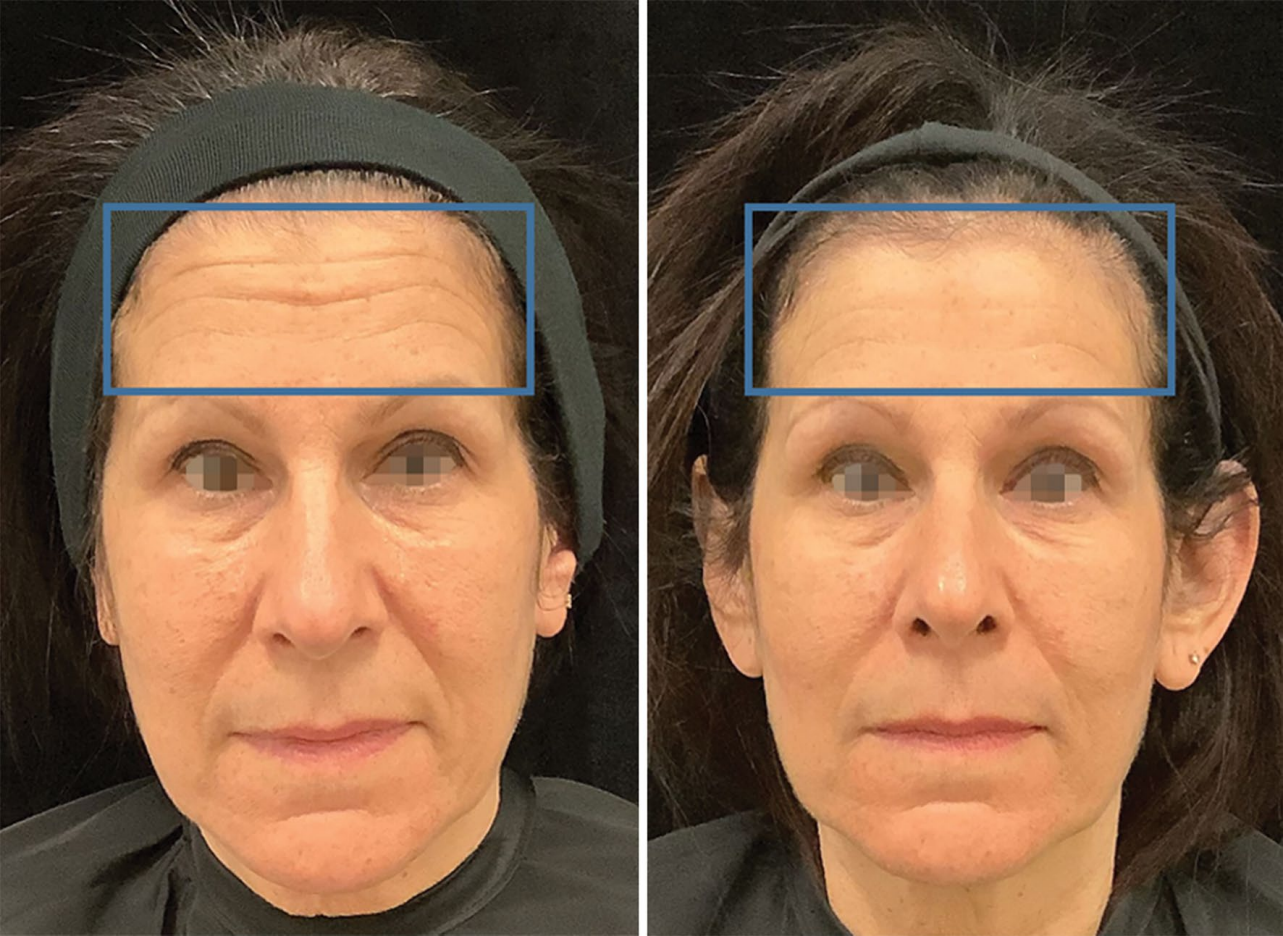
Representative photos of a 60‐year‐old patient treated with simultaneous application of RF + TUS show a visible and clinically rejuvenated face (forehead) with smoothed lines, digital photograph taken before the treatment (left) and at the 3‐month follow‐up visit (right).

Digital photographs of the RF + TUS group showed both a decrease in rhytids and a tighter skin (Figure 6). The aesthetic improvements in the treated subjects were evaluated using GAIS and FWES scales.

The subjects in Group A (RF + TUS) showed a significant GAIS improvement in facial appearance with smoothed lines. The most prominent improvement of 1.22 ± 0.38 points to 1.88 ± 0.19 was observed in the RF + TUS group at 3 months. In the RF only (Group B), there was a GAIS score improvement of 0.66 ± 0.33 to 0.77 ± 0.19 points at 3 months, with no changes observed by the evaluators.

Subjects treated with RF + TUS (group A) showed significant improvement from an average baseline FWES value of 5.33 ± 0.33 points to 3.11 ± 0.19 at 3‐month follow‐up visits. In group B (RF only), there was no significant decrease in wrinkle severity with an average baseline FWES value of 4.44 ± 0.19 and 4.11 ± 0.38 points at 3 months.

### 3.3 Subject Satisfaction and Therapy Comfort

The subjects from Group A found the treatment (RF + TUS) very comfortable (4.0/5) with mild discomfort (2.3/10) and reported a high satisfaction (4.0/5) with the treatment result, improvement in skin laxity, and overall skin appearance at 3 months. Subjects from Group B (RF only) were less comfortable with their treatment (3.3/5), found it to be moderately painful (5.3/10) and were less satisfied with their overall appearance (3.4/5).

### 3.4 Safety and Adverse Events

There were no adverse events reported throughout the study, and no downtime was experienced by any subject from both treated groups (A and B) due to the treatment.

## 4 Discussion

This clinical study evaluated the clinical efficacy and safety of the combined therapy of RF and TUS for facial rejuvenation through changes in facial skin tissue related to the levels of HA. The results obtained from this study support the use of monopolar RF combined with TUS as an effective technique for facial rejuvenation. This was evidenced by both quantitative histological assessments and qualitative visual evaluations carried out.

The histological analysis of the HA‐stained area revealed statistically significant (*p* < 0.05) changes occurring within the facial skin following treatment. In Group A, which received the simultaneous RF + TUS treatment, an average increase of 48.65% was observed at 1 month compared to baseline. This substantial change signified a robust initial response to the combined therapy. Interestingly, the treatment effect exhibited a progressive improvement, peaking at 3 months posttreatment with an even larger increase of 67.69% in the HA‐stained area. This pronounced enhancement at 3 months suggests a sustained and cumulative effect of the RF + TUS treatment on skin rejuvenation (see Figure 5). In contrast, Group B, which underwent the standalone RF treatment regimen, did not exhibit the same level of improvement. The average increases in HA‐stained area observed at 1 and 3 months were statistically insignificant, corresponding to only 6.76% and 9.56% increases, respectively. There were no changes in the control samples.

This disparity in outcomes demonstrates that the combined treatment modality (RF + TUS) exerts a more pronounced effect on the skin's extracellular matrix. The absence of any HA‐containing substances applied suggests that the increased HA levels can be attributed to the study treatment. HA production is proposed to result from the combined effects of RF‐generated thermal energy and TUS's mechanical energy, which together promote fibroblast activity in the dermis [12, 14, 15, 20, 21, 22, 23]. It was also observed from this present study that the treatment peaked at 3 months. This is consistent with previous research that has shown that structural remodeling in skin tissue after mechanical and thermal stimulation occurs approximately three months after treatment [21, 24, 25, 26]. Additionally, findings from previous animal studies using this combination therapy showed that the use of RF + TUS led to a significant increase in HA levels in porcine skin samples, while RF alone did not have the same effect. This suggests the crucial role of TUS in altering tissue composition for increased HA production. However, further research examining the standalone effect of TUS is recommended to gain a clearer understanding of this mechanism.

Furthermore, the digital photographs taken for the RF + TUS group provided additional supportive evidence of the combination treatment's efficacy. These photographs (Figure 6) were carefully assessed by three independent evaluators using the GAIS and FWES scales revealing a decrease in rhytids (wrinkles), improvement in skin laxity and overall facial appearance, which aligns with the quantitative data obtained from the HA‐stained area measurements. These qualitative improvements validate the clinical relevance of the study's findings that the combination therapy not only induces structural changes at the cellular level but also translates into visible enhancements in facial appearance. The role of HA in maintaining skin hydration and volume has been investigated by Zhang et al. [3] Similarly, other researchers have also reported that HA acts as a natural moisturizer and provides support to the main fibers that form the extracellular matrix, contributing to skin elasticity and firmness [27, 28, 29]. These previous investigations correspond with the clinical observations of reduced wrinkles, increased skin laxity, firmness, and a refreshed overall appearance reported by the Group A (RF + TUS) subjects in this study.

Another important aspect of this study is the safety and tolerability profile of the combined RF and TUS therapy. The subjects from Group A found the treatment (RF + TUS) very comfortable and reported high satisfaction with the treatment results, further enhancing the clinical feasibility and attractiveness of this approach for facial rejuvenation. Subjects from Group B (RF only) were less comfortable with their treatment and found their therapy moderately painful. This suggests that comfort levels were enhanced by the ultrasound component of the combination therapy. Similar to the previous studies, during this investigation, there were no treatment‐related adverse events. This further underscores the safety of the combined RF + TUS treatments used for skin rejuvenation, and the trust patients can place in the safety profile of the therapy.

Numerous studies on RF modalities used for facial rejuvenation emphasize their skin‐tightening effects but generally do not indicate active promotion of the body's natural HA production within the dermal layers [15, 24]. The results of this study suggest that the conclusions drawn from previous veterinary studies regarding the effect of the simultaneous application of RF and TUS on natural HA enhancement are applicable to human skin as well. TUS waves are expected to activate cell receptors, causing changes in gene expression and protein synthesis within the fibroblasts. This modification enhances the permeability of cells and enables more effective transmission of RF energy to deeper skin layers, without causing any discomfort to the patient [12, 13]. By increasing the levels of HA in the dermis, skin hydration is improved, and the overall rejuvenation effect is completed.

Nonetheless, a few limitations of our study must be acknowledged. The sample size was small, and the study duration was limited to a 3‐month follow‐up period. Additionally, collagen and elastin content, which are the crucial extracellular matrix components involved in skin aging, were not analyzed following the combined RF + TUS application, as it was beyond the scope of this study. Future research is recommended to evaluate the effects of combined RF + TUS energies on dermal collagen and elastin, as well as to analyze TUS as a standalone treatment to compare its role in enhancing HA levels. Further investigations with larger sample sizes and extended follow‐up periods may provide a more comprehensive understanding of the long‐term efficacy and durability of the observed effects of this combination therapy.

## 5 Conclusion

Overall, this study has shown that the combination treatment of RF + TUS has a more pronounced and sustained effect on facial rejuvenation compared to RF alone. The combination of both monopolar RF and TUS enhanced the deposition of HA within the facial skin, leading to improved skin quality and texture. The treatment was well tolerated by all the subjects, and no adverse events were reported. By harnessing the body's natural ability to produce HA, this novel noninvasive technology offers an effective and safe alternative to invasive procedures such as dermal fillers or surgical interventions. In addition, the targeted nature of the treatments ensures that the production of HA is concentrated in specific areas, allowing for precise facial rejuvenation and improved skin quality. For these reasons, the findings from this study will have significant implications for the field of aesthetic medicine and antiaging treatments.

## Ethics Statement

Author declares that human ethics approval was obtained for this study.

## Conflicts of Interest

The author declares no conflicts of interest.

## Data Availability

The data that supports the findings of this study are available in the Supporting Information of this article.

## References

[1] A. Huynh and R. Priefer , “Hyaluronic Acid Applications in Ophthalmology, Rheumatology, and Dermatology,” Carbohydrate Research 489 (2020): 107950, 10.1016/j.carres.2020.107950. 32070808

[2] M. A. Keen , “Hyaluronic Acid in Dermatology,” Skinmed 15, no. 6 (2017): 441–448. 29282181

[3] W. Zhang , H. Mu , A. Zhang , et al., “A Decrease in Moisture Absorption‐Retention Capacity of N‐Deacetylation of Hyaluronic Acid,” Glycoconjugate Journal 30, no. 6 (2012): 577–583, 10.1007/s10719-012-9457-3. 23224991

[4] J. H. Oh , Y. K. Kim , J. Y. Jung , et al., “Intrinsic Aging‐ and Photoaging‐Dependent Level Changes of Glycosaminoglycans and Their Correlation With Water Content in Human Skin,” Journal of Dermatological Science 62, no. 3 (2011): 192–201, 10.1016/j.jdermsci.2011.02.007. 21477996

[5] S. Zhang and E. Duan , “Fighting Against Skin Aging: The Way From Bench to Bedside,” Cell Transplantation 27, no. 5 (2018): 729–738, 10.1177/0963689717725755. 29692196 PMC6047276

[6] J. R. Thomas , “Update on Facial Skin Rejuvenation Technology,” Facial Plastic Surgery Clinics of North America 28, no. 1 (2020): fsc001, 10.1016/j.fsc.2019.10.001. 31779947

[7] R. Murthy , J. C. P. Roos , and R. A. Goldberg , “Periocular Hyaluronic Acid Fillers: Applications, Implications, Complications,” Current Opinion in Ophthalmology 30, no. 5 (2019): 395–400, 10.1097/ICU.0000000000000595. 31261189

[8] S. Vasvani , P. Kulkarni , and D. Rawtani , “Hyaluronic Acid: A Review on Its Biology, Aspects of Drug Delivery, Route of Administrations and a Special Emphasis on Its Approved Marketed Products and Recent Clinical Studies,” International Journal of Biological Macromolecules 151 (2020): 1012–1029, 10.1016/j.ijbiomac.2019.11.066. 31715233

[9] P. Wongprasert , C. A. Dreiss , and G. Murray , “Evaluating Hyaluronic Acid Dermal Fillers: A Critique of Current Characterization Methods,” Dermatologic Therapy 35, no. 6 (2022): e15453, 10.1111/dth.15453. 35293660 PMC9285697

[10] D. Duncan , J. Bernardy , N. Hodkovicova , and J. Masek , Increased Levels of Hyaluronic Acid in Skin After Monopolar Radiofrequency and Targeted Ultrasound Treatment: Porcine Animal Study (American Society for Laser Medicine and Surgery (ASLMS), 2022).

[11] S. Chilukuri , D. Denjean , and L. Fouque , “Treating Multiple Body Parts for Skin Laxity and Fat Deposits Using a Novel Focused Radiofrequency Device With an Ultrasound Component: Safety and Efficacy Study,” Journal of Cosmetic Dermatology 16, no. 4 (2017): 476–479, 10.1111/jocd.12448. 29125214

[12] S. P. Bohari , L. M. Grover , and D. W. Hukins , “Pulsed Low‐Intensity Ultrasound Increases Proliferation and Extracelluar Matrix Production by Human Dermal Fibroblasts in Three‐Dimensional Culture,” Journal of Tissue Engineering 6 (2015): 5777, 10.1177/2041731415615777. PMC4674020 26668710

[13] K. Minkis and M. Alam , “Ultrasound Skin Tightening,” Dermatologic Clinics 32, no. 1 (2014): 71–77, 10.1016/j.det.2013.09.001. 24267423

[14] R. D. Gentile , B. M. Kinney , and N. S. Sadick , “Radiofrequency Technology in Face and Neck Rejuvenation,” Facial Plastic Surgery Clinics of North America 26, no. 2 (2018): 123–134, 10.1016/j.fsc.2017.12.003. 29636146

[15] A. S. Levy , R. T. Grant , and K. O. Rothaus , “Radiofrequency Physics for Minimally Invasive Aesthetic Surgery,” Clinics in Plastic Surgery 43, no. 3 (2016): 551–556, 10.1016/j.cps.2016.03.013. 27363769

[16] S. R. Mehta‐Ambalal , “Neocollagenesis and Neoelastinogenesis: From the Laboratory to the Clinic,” Journal of Cutaneous and Aesthetic Surgery 9, no. 3 (2016): 145–151, 10.4103/0974-2077.191645. 27761083 PMC5064677

[17] R. Wong , S. Geyer , W. Weninger , J. C. Guimberteau , and J. K. Wong , “The Dynamic Anatomy and Patterning of Skin,” Experimental Dermatology 25, no. 2 (2016): 92–98, 10.1111/exd.12832. 26284579

[18] D. Bertossi , A. Sbarbati , R. Cerini , et al., “Hyaluronic Acid: In Vitro and In Vivo Analysis, Biochemical Properties and Histological and Morphological Evaluation of Injected Filler,” European Journal of Dermatology 23, no. 4 (2013): 449–455, 10.1684/ejd.2013.2059. 24052368

[19] A. Fernandez‐Flores , “Regional Variations in the Histology of the Skin,” American Journal of Dermatopathology 37, no. 10 (2015): 737–754, 10.1097/DAD.0000000000000353. 26381022

[20] H. J. Lee , S. R. Seo , M. S. Yoon , J. Y. Song , E. Y. Lee , and S. E. Lee , “Microneedle Fractional Radiofrequency Increases Epidermal Hyaluronan and Reverses Age‐Related Epidermal Dysfunction,” Lasers in Surgery and Medicine 48, no. 2 (2016): 140–149, 10.1002/lsm.22420. 26415023

[21] P. F. Meyer , F. K. B. A. Silva , A. C. S. da Costa , et al., “Radiofrequency Treatment Induces Fibroblast Growth Factor 2 Expression and Subsequently Promotes Neocollagenesis and Neoangiogenesis in the Skin Tissue,” Lasers in Medical Science 32, no. 8 (2017): 1727–1736, 10.1007/s10103-017-2238-2. 28569344

[22] H. Park , E. Kim , J. Kim , Y. Ro , and J. Ko , “High‐Intensity Focused Ultrasound for the Treatment of Wrinkles and Skin Laxity in Seven Different Facial Areas,” Annals of Dermatology 27, no. 6 (2015): 688–693, 10.5021/ad.2015.27.6.688. 26719637 PMC4695420

[23] J. Y. Lee , D. J. Min , W. Kim , B. H. Bin , K. Kim , and E. G. Cho , “Non Pharmacological High‐Intensity Ultrasound Treatment of Human Dermal Fibroblasts to Accelerate Wound Healing,” Scientific Reports 11, no. 1 (2021): 2465, 10.1038/s41598-021-81878-1. 33510199 PMC7844265

[24] R. A. Weiss , “Noninvasive Radio Frequency for Skin Tightening and Body Contouring,” Seminars in Cutaneous Medicine and Surgery 32, no. 1 (2013): 9–17. 24049924

[25] D. H. Ahn , R. S. Mulholland , D. Duncan , and M. Paul , “Non‐Excisional Face and Neck Tightening Using a Novel Subdermal Radiofrequency Thermo‐Coaugulative Device,” Journal of Cosmetics, Dermatological Sciences and Applications 1, no. 4 (2011): 141–146, 10.4236/jcdsa.2011.14021.

[26] B. Hantash , A. Ubeid , H. Chang , R. Kafi , and B. Renton , “Bipolar Fractional Radiofrequency Treatment Induces Neoelastogenesis and Neocollagenesis,” Lasers in Surgery and Medicine 41 (2009): 1–9, 10.1002/lsm.20731. 19143021

[27] Y. Qiu , Y. Ma , Y. Huang , S. Li , H. Xu , and E. Su , “Current Advances in the Biosynthesis of Hyaluronic Acid With Variable Molecular Weights,” Carbohydrate Polymers 269 (2021): 118320, 10.1016/j.carbpol.2021.118320. 34294332

[28] V. Thulabandu , D. Chen , and R. P. Atit , “Dermal Fibroblast in Cutaneous Development and Healing,” WIREs Developmental Biology 7, no. 2 (2018): e307, 10.1002/wdev.307. PMC5814349 29244903

[29] E. Papakonstantinou , M. Roth , and G. Karakiulakis , “Hyaluronic Acid: A Key Molecule in Skin Aging,” Dermato‐Endocrinology 4, no. 3 (2012): 253–258, 10.4161/derm.21923. 23467280 PMC3583886

